# TWEAK/Fn14 Signaling Axis Mediates Skeletal Muscle Atrophy and Metabolic Dysfunction

**DOI:** 10.3389/fimmu.2014.00018

**Published:** 2014-01-27

**Authors:** Shuichi Sato, Yuji Ogura, Ashok Kumar

**Affiliations:** ^1^Department of Anatomical Sciences and Neurobiology, University of Louisville School of Medicine, Louisville, KY, USA

**Keywords:** TWEAK, Fn14, skeletal muscle, atrophy, oxidative phosphorylation

## Abstract

Tumor necrosis factor-like weak inducer of apoptosis (TWEAK) through binding to its receptor fibroblast growth factor inducible 14 (Fn14) has been shown to regulate many cellular responses including proliferation, differentiation, apoptosis, inflammation, and fibrosis, under both physiological and pathological conditions. Emerging evidence suggests that TWEAK is also a major muscle wasting cytokine. TWEAK activates nuclear factor-κB signaling and proteolytic pathways such as ubiquitin–proteasome system, autophagy, and caspases to induce muscle proteolysis in cultured myotubes. Fn14 is dormant or expressed in minimal amounts in normal healthy muscle. However, specific atrophic conditions, such as denervation, immobilization, and starvation stimulate the expression of Fn14 leading to activation of TWEAK/Fn14 signaling and eventually skeletal muscle atrophy. TWEAK also causes slow- to fast-type fiber transition in skeletal muscle. Furthermore, recent studies suggest that TWEAK diminishes mitochondrial content and represses skeletal muscle oxidative phosphorylation capacity. TWEAK mediates these effects through affecting the expression of a number of genes and microRNAs. In this review article, we have discussed the recent advancements toward understanding the role and mechanisms of action of TWEAK/Fn14 signaling in skeletal muscle with particular reference to different models of atrophy and oxidative metabolism.

## Introduction

Skeletal muscle is the largest tissue of the human body, which provides posture, ensures basic functions such as locomotion and respiration, and plays a vital role in whole body metabolism. Skeletal muscle is also one of the most highly plastic tissues in the body. A number of extracellular and intracellular signals have now been identified, which cause physiological adaptations including enhanced substrate metabolism, mitochondrial biogenesis, angiogenesis, muscle growth, and regeneration (1, 2). For example, exercise training causes improvement in muscle mass and contractility, and metabolic function resulting in enhanced force generation capacity and resistance to fatigability. Conversely, inactivity and many chronic disease states result in loss of skeletal muscle mass and metabolic dysfunction (3, 4). Maintenance of skeletal muscle mass and function are prerequisites for whole body health throughout life.

Skeletal muscle mass is maintained through a delicate balance between protein synthesis and degradation. Resistance exercise, hormones, and nutritional uptake increase rate of protein synthesis resulting in increased muscle mass (5). Insulin-like growth factor-1 (IGF-1)/phosphatidylinositol 3-kinase (PI3K)/Akt/mTOR is one of the most important signaling pathways, which increases protein synthesis leading to skeletal muscle hypertrophy (6, 7). This pathway also inhibits muscle protein degradation through distinct mechanisms (8, 9). By contrast, many catabolic stimuli increase the activity of various signaling intermediates, such as extracellular-regulated kinase 1/2 (ERK1/2), c-Jun-N-terminal kinase (JNK), p38 mitogen-activated protein kinase (MAPK), AMP-activated protein kinase (AMPK), and TNF receptor-associated factors (TRAFs) and transcription factors, such as nuclear factor-κB (NF-κB), activator protein 1 (AP1), p53, FoxO1, and FoxO3a resulting in the activation of ubiquitin–proteasome system (UPS) and autophagy–lysosomal system (ALS), the two major proteolytic mechanisms in skeletal muscle (1, 10, 11). Recent studies also suggest that activating transcription factor 4 (ATF4), growth arrest and DNA damage-inducible 45α (GADD45α), histone deacetylase 4 (HDAC4), and myogenin mediate skeletal muscle atrophy under specific conditions (12–14). Furthermore, changes in the mitochondrial content, integrity, and function play a critical role in regulation of skeletal muscle mass and function (15, 16). The biogenesis of new mitochondria and clearance of defunct mitochondria are essential to meet cellular energy demand especially during endurance exercise and to protect from many chronic diseases such as diabetes, heart failure, obesity, and cancer (17, 18). Impairment in mitochondrial function is also an important facet of aged skeletal muscle. However, it is not yet clear whether dysfunctional mitochondria are a cause or consequence of aged skeletal muscle (19).

Classical proinflammatory cytokines, such as tumor necrosis factor-α (TNFα), interleukin-1β (IL-1β), IL-6, and interferon-γ have been suggested as important mediators of catabolic response (protein loss and insulin resistance), contractile dysfunction, and disruption of muscle regenerative ability in many chronic disease states (20–24). Although, neutralization of some of these cytokines attenuates muscle wasting, blocking their activity failed to preserve skeletal muscle mass in some conditions where even their abundance was significantly elevated suggesting that there are additional mediators of muscle wasting (24). TNF-like weak inducer of apoptosis (TWEAK), a member of the TNF superfamily, has recently been identified as an important mediator of skeletal muscle wasting and metabolic dysfunction. While the levels of several classical muscle wasting cytokines are increased in various chronic disease states, there is no evidence about their potential role in the loss of muscle mass in disuse conditions (e.g., denervation, immobilization, and unloading). Our studies have shown that TWEAK/Fn14 system also mediates skeletal muscle wasting in disuse conditions and even in response to starvation. Moreover, increased levels of TWEAK inhibit skeletal muscle regenerative capacity through affecting self-renewal of satellite cells and proliferation, fusion, and differentiation of myoblasts into multinucleated myotubes both *in vitro* and *in vivo* (25–29). The role of TWEAK/Fn14 signaling in regulation of muscle progenitor cell biology has been discussed in another article in this issue (30). We have focused our review article to discuss the recent advancement toward understanding the role and mechanisms of action of TWEAK in adult skeletal muscle.

## Brief Overview TWEAK/Fn14 Dyad

TWEAK is a member of TNF super family (TNFSF) cytokines, which is initially synthesized as type II transmembrane protein (249 amino acids) similar to TNF-α. However, membrane-bound TWEAK is cleaved to its soluble form (156 amino acids) by furin, a calcium-dependent serine endoprotease (31–33). Fn14, a type I transmembrane protein, was first recognized by differential display analysis and later identified as the unique TWEAK receptor (34–36). Fn14 is the smallest member (102 amino acids) of TNF receptor super family (TNFRSF), which is expressed at relatively low levels in normal healthy tissues except in progenitor cells. However, the expression of Fn14 is drastically increased in response to tissue injury and various pathological conditions (31, 33). Similar to other members of TNFRSF, the cytoplasmic domain of Fn14 contains a TRAF binding site, which allows downstream signaling upon stimulation by TWEAK (37). By contrast, Fn14 lacks a death domain and does not show any association with death-inducing signaling complex (33). Furthermore, some studies suggest that Fn14 can also function by clustering themselves on the cell membrane under low TWEAK condition. TWEAK/Fn14 system plays an important role in tissue homeostasis through regulating cell survival, proliferation, migration, and angiogenesis (31, 33). Conversely, aberrant regulation of TWEAK/Fn14 signaling causes many pathological consequences including cancer, rheumatoid arthritis, systemic lupus erythematous, multiple sclerosis, skeletal muscle wasting, and cardiac dysfunction and failure (31).

**Figure 1:**
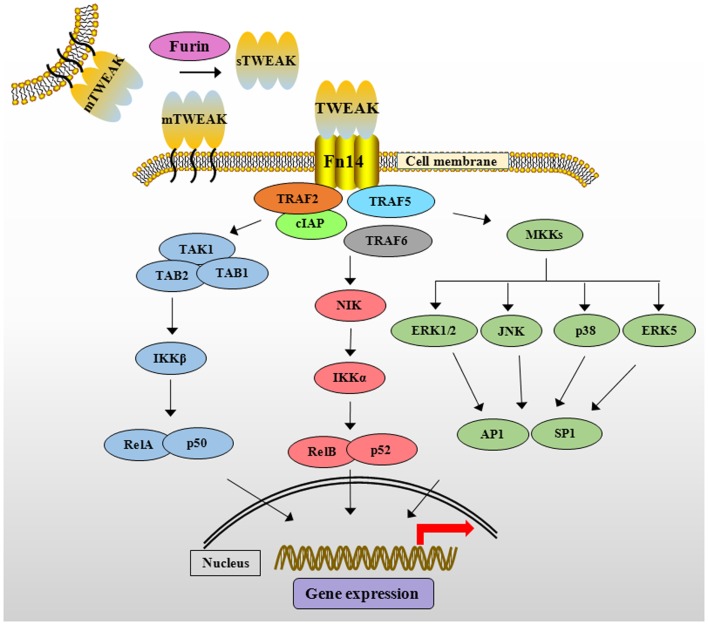
**The TWEAK/Fn14 signaling cascade**. TWEAK cytokine is initially synthesized as membrane-anchored protein (mTWEAK). mTWEAK is then cleaved by furin into soluble form of TWEAK (sTWEAK). Binding of trimeric mTWEAK or sTWEAK to Fn14 receptor leads to the recruitment of cIAPs and various TRAFs resulting in the activation of multiple downstream signaling cascades. TWEAK stimulates canonical NF-κB pathway through activation of TAK1 and IKKβ and phosphorylation and degradation of IκB protein. TWEAK activates non-canonical NF-κB pathway through activation of NIK and IKKα leading to proteolytic processing of p100 protein into p52. TWEAK-mediated signaling also causes the activation of various MKKs, which phosphorylate ERK1/2, ERK5, JNK, and p38MAPK resulting in the activation of downstream transcription factors such as AP1 and SP1. TWEAK, TNF-like weak inducer of apoptosis; Fn14, fibroblast growth factor inducible 14; cIAP, cellular inhibitor of apoptosis; TRAF, TNF receptor-associated factor; TAK1, transforming growth factor β-activated kinase 1, TAB, TAK1 binding protein; IKK, IκB kinase β; IκB, inhibitor of nuclear factor of kappa B; NIK, NF-κB-inducing kinase; MAPK, mitogen-activated protein kinase; MKKs, mitogen-activated protein kinase kinases; ERK, extracellular signal-regulating kinase; JNK, c-Jun N-terminal kinase; AP1, activator protein 1; SP1, specificity protein 1.

Like other members of TNFSF, TWEAK mediates unique and context-dependent pleiotropic effects. In contrast to TNF-α, TWEAK attenuates the transition from innate to adaptive immunity by suppressing the production of interferon-γ and IL-12 cytokines (38). Moreover, TWEAK activates multiple intracellular pathways such as MAPK, PI3K/Akt, and canonical and non-canonical NF-κB signaling in various cell types (39–42). Binding of TWEAK leads to the formation of TRAF2/cIAP1 (cellular inhibitor of apoptosis protein 1) complex at Fn14 cytoplasmic domain that results in the activation of various signaling proteins including TRAF6, transforming growth factor-β activated kinase1 (TAK1), I kappa β kinase (IKK), NF-κB-inducing kinase (NIK), and MAPKs, which regulate expression of several molecules involved in various cellular responses (Figure 1) (33, 39, 43–45). Moreover, the activation of TWEAK/Fn14 is coupled with TNFα–TNFR1 signaling. For example, the activation of TWEAK/Fn14 signaling sensitizes TNF-α signaling by exhausting cytosolic TRAF2/cIAP1 complex (44).

## TWEAK Causes Atrophy in Cultured Myotubes

C2C12 as well as mouse primary myoblasts differentiate into myotubes upon incubation in low serum conditions. These cultured myotubes serve as an excellent model to study atrophy in response to exogenously added molecules. We have previously reported that treatment of cultured myotubes with even low concentrations of TWEAK protein causes severe atrophy (46, 47). Multiple studies have shown that skeletal muscle atrophy is associated with rapid degradation of selective muscle proteins including myosin heavy chain (MyHC) (48–50). Consistent with effects on myotube size, addition of TWEAK led to significant reduction in overall protein content and levels of MyHC in cultured myotubes (46, 47). Moreover, at equimolar concentration, TWEAK was found to be more potent in inducing MyHC degradation compared with its structural homolog TNF-α suggesting that TWEAK is a potent muscle wasting cytokine (47).

The UPS is one of the most important proteolytic systems that cause selective degradation of muscle structural proteins (1, 11, 51). Two muscle-specific E3 ubiquitin ligases, muscle RING-finger 1 (*MuRF1*) and muscle atrophy F-box (*MAFBx*; also known as Atrogin-1) are the key enzymes of the UPS involved in degradation of muscle proteins. Levels of both MuRF1 and MAFBx are drastically increased in atrophying skeletal muscle, where disruption of these genes in mice preserves skeletal muscle mass in many catabolic conditions, such as hind limb unloading, cast immobilization, and denervation (1, 11, 52). ALS is another proteolytic mechanism that contributes to degradation of muscle proteins and cellular organelles in skeletal muscle in response to various atrophic stimuli. In autophagy, cytosolic proteins and organelles are sequestered by an isolation-membrane structure to form autophagosome. The autophagosome then fuses with lysosome, where the autophagosome is degraded to amino acid or peptide (53). Autophagy constitutively takes place at lower levels in most cells under normal condition and is an essential component of homeostasis machinery in different tissues including skeletal muscle (53, 54). However, excessive activation of autophagy leads to muscle wasting in many conditions such as starvation and denervation (8, 55).

Our studies have shown that TWEAK increases the gene expression of MuRF1 and MAFBx and stimulates the conjugation of ubiquitin with MyHC in cultured myotubes (46, 47). Furthermore, TWEAK also induces the expression of several components of ALS and activates caspases in cultured myotubes (46). Indeed, inhibition of the activity of either UPS, autophagy, or caspases significantly reduced TWEAK-induced degradation of MyHC and myotube atrophy suggesting that TWEAK induces muscle proteolysis through coordinated activation of multiple proteolytic systems (46).

**Figure 2:**
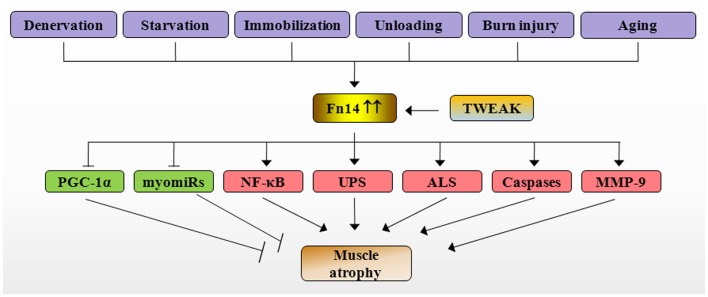
**Mechanisms of action of TWEAK/Fn14 system in skeletal muscle atrophy**. Specific catabolic conditions such as denervation, starvation, immobilization, unloading, burn injury, and aging augments the expression of Fn14 resulting in stimulation of TWEAK/Fn14 signaling in skeletal muscle. TWEAK-Fn14 signaling represses the levels of PGC-1α and myomiRs and increases the activation of NF-κB, UPS, ALS, caspases, and MMP-9, which eventually cause loss of skeletal muscle mass. Fn14, fibroblast growth factor inducible 14; TWEAK, TNF-like weak inducer of apoptosis; PGC-1α, peroxisome proliferator-activated receptor-gamma coactivator 1α; MMP-9, matrix metalloproteinase-9; NF-κB, nuclear factor-kappa B; UPS, ubiquitin–proteasome system; ALS, autophagy–lysosome system; myomiRs, muscle microRNAs.

NF-κB is a major proinflammatory transcription factor, which is strongly linked to skeletal muscle wasting not only in chronic diseases but also in disuse conditions (10). Inhibition of NF-κB has been found to rescue skeletal muscle atrophy in response to cytokines, tumor growth, denervation, and unloading (10, 56). By contrast, forced activation of NF-κB is sufficient to cause severe muscle wasting in mice (56). NF-κB induces muscle atrophy through augmenting the expression of MuRF1 and several other components of UPS (10). It has been recently reported that MuRF1 causes breakdown of MyHC and other components of the thick filament of the sarcomere during atrophy (57, 58). Our studies have shown that TWEAK causes sustained activation of NF-κB. Furthermore, inhibition of NF-κB through pharmacological or molecular approaches prevents the degradation of MyHC and atrophy in cultured myotubes (46, 47, 59, 60). Although, TWEAK did not inhibit protein synthesis, it inhibited the activation of PI3K/Akt/mTOR signaling pathway in cultured myotubes (47). Similarly, TWEAK has also been found to inhibit the insulin-induced Akt phosphorylation in hepatocytes (61). Since Akt signaling inhibits the activity of FoxO family of transcription factors, which are required for the expression of MuRF1 and MAFBx (1), it is likely that the inhibition of Akt signaling is another mechanism by which TWEAK augments the gene expression of MuRF1 and MAFBx in cultured myotubes. Collectively, these observations suggest that TWEAK causes atrophy in cultured myotubes through the activation of NF-κB and various proteolytic systems such as UPS, ALS, and caspases (Figure 2).

The effect of TWEAK on global gene expression and microRNAs (miRs) in cultured myotubes has also been investigated. Pathway analysis of microarray gene expression data suggested that TWEAK affects the activation of several toxic pathways including those involved in initiation and manifestation of fibrosis, oxidative stress, and mitochondrial dysfunction in skeletal muscle (62). For example, TWEAK reduces the expression of structural molecules (e.g. MyHC, TCap, and ankyrin repeat domain 2), metabolic enzymes (e.g., phosphoglycerate mutase 2), and signaling proteins (e.g., Notch1 and TRAF6) in C2C12 myotubes. By contrast, TWEAK induces the expression of multiple components of NF-κB signaling pathways and expression of matrix metalloproteinase-9 (MMP-9) in myotubes (62). Recently, a few muscle-specific miRs such as miR-1, miR-133a, miR-133b, and miR-206 (also called myomiRs) have been identified, which are essential for muscle progenitor cell proliferation, differentiation, and maintenance. Expression of miR-1 and miR-133a in embryonic stem cells and other non-muscle cell types showed that they promote the differentiation into the skeletal muscle lineage. Unlike other myomiRs, which are also expressed in cardiac tissues, miR-133b and miR-206 are predominantly expressed in skeletal muscle (63). Interestingly, microarray analysis of miRs has shown that TWEAK reduces the expression levels of miR-1, miR-133a, miR-133b, and miR-206 in cultured myotubes (62). Recent studies have demonstrated that myogenic transcription factors, such as myocyte enhancer factor 2c (MEF2c) and MyoD control the expression of myomiRs in skeletal and cardiac muscles (63). Interestingly, TWEAK diminishes the levels of both MEF2c and MyoD (25, 62) suggesting that reduced expression of myomiRs could be a result of diminished abundance of MEF2c and MyoD in TWEAK-treated myotubes. TWEAK also regulates the expression of a number of other miRs, which are involved in distinct biological responses. Using differentially regulated miR data set, we generated a network of pathways from differentially regulated genes in cDNA microarray data set. This analysis showed that miRs network overlaps with mRNA networks suggesting that miRs may play important roles in the regulation of gene expression and skeletal muscle structure and function in response to TWEAK (62).

## TWEAK Causes Skeletal Muscle Atrophy *In vivo*

*In vivo* effects of TWEAK on skeletal muscle were first investigated by systemic and chronic administration of soluble TWEAK proteins in wild-type mice. Chronic administration of TWEAK in mice led to a significant reduction in whole body weight, individual muscle mass, and fiber-cross-sectional area (47). Furthermore, transgenic (Tg) overexpression of full-length TWEAK using muscle creatine kinase (MCK) promoter resulted in smaller muscle size and neonatal lethality in mice (47). TWEAK-Tg mice, which survived due to relatively low expression of TWEAK (four to sixfolds higher than littermate controls) showed muscle atrophy and interstitial fibrosis at around 4–6 months of age (43). Consistent with *in vitro* studies, transcript levels of MuRF1 and ubiquitination of MyHC were significantly elevated in skeletal muscle of TWEAK-Tg mice compared with their littermate controls. By contrast, there was no significant difference in mRNA levels of MAFBx in skeletal muscle of wild-type and TWEAK-Tg mice indicating that TWEAK causes muscle atrophy *in vivo* through augmenting the expression of MuRF1 (43, 62). Furthermore, the gene expression of several components of NF-κB signaling pathway and DNA-binding activity of NF-κB were significantly increased in skeletal muscle of TWEAK-Tg mice (43). These findings suggest that the activation of NF-κB pathway is one of the important mechanisms for TWEAK-induced muscle proteolysis (Figure 2).

Accumulating evidence suggests that peroxisome proliferator-activated receptor γ (PPAR-γ) coactivator 1α (PGC-1α) plays a key role in preserving skeletal muscle mass and mitochondrial content in atrophic conditions (64). Levels of PGC-1α are repressed in multiple atrophy conditions whereas muscle-specific overexpression of PGC-1α rescues the loss of skeletal muscle mass in catabolic states (65, 66). Our recent studies demonstrate that TWEAK represses the expression of PGC-1α in cultured myotubes (67). Overexpression of PGC-1α inhibits TWEAK-induced atrophy, NF-κB activation, and expression of MAFBx and MuRF1 in cultured myotubes. Moreover, progressive muscle atrophy observed in TWEAK-Tg mice is significantly attenuated in TWEAK-PGC-1α double Tg mice suggesting that PGC-1α plays an important role in TWEAK-induced muscle atrophy (67).

Another potential mechanism by which TWEAK causes muscle atrophy is through deregulation of matrix metalloproteinases (MMPs). Protein levels as well as enzymatic activity of MMP-9 are significantly elevated in skeletal muscle of TWEAK-Tg mice (59, 62). Since, muscle atrophy in response to chronic administration of TWEAK is rescued to some extent in *Mmp9*-knockout (KO) mice, it is likely that MMP-9 is involved in TWEAK-induced skeletal muscle wasting *in vivo*. Elevated levels of MMP-9 can potentially induce muscle atrophy through breakdown of extracellular matrix and proteolytic activation of other catabolic cytokines (59). In agreement with microarray data in TWEAK-treated myotubes, we have found that the levels of many structural molecules such as MyHC, TCap, and ankyrin repeat domain 2 and myomiRs were reduced in skeletal muscle of TWEAK-Tg mice suggesting that TWEAK also causes atrophy through repression of specific muscle proteins (62).

## TWEAK/Fn14 System Mediates Muscle Atrophy in Catabolic Conditions

Muscle atrophy can be observed in various physiological and pathophysiological conditions such as bed-rest, space flight, aging, cancer, severe burn injury, starvation, high dose glucocorticoid therapy, and neurotmesis (2). Our group and others have recently investigated whether the gene expression of TWEAK or Fn14 is affected in skeletal muscle in different atrophy conditions. Interestingly, the gene expression of Fn14 is dramatically induced in skeletal muscle in disuse conditions such as denervation and immobilization (43) and in response to starvation (48). Wu et al. studied the global gene expression in skeletal muscle of mice in response to hind limb suspension, a model to elicit unloading-induced skeletal muscle atrophy. Microarray and quantitative real-time PCR assays showed that the gene expression of Fn14 is significantly increased in skeletal muscle of mice (68). Moreover, a recent study has also demonstrated that the levels of TWEAK and Fn14 are increased in human skeletal muscle in response to severe burn injury (69). By contrast, mRNA levels of TWEAK and Fn14 remained unaffected in skeletal muscle of mice in response to high doses of glucocorticoids (43) and in skeletal muscle of patients with chronic obstructive pulmonary disease (70) indicating that TWEAK/Fn14 system may not be the mediator of muscle atrophy in all conditions.

The physiological role of TWEAK/Fn14 system in muscle atrophy has been investigated through a series of experiments using denervated hind limb muscle as a model of neurotmesis (43). Levels of Fn14 protein are dramatically increased upon denervation regardless of muscle fiber type (43). Denervation-induced loss of skeletal muscle mass and fiber cross-sectional-area were significantly exaggerated in TWEAK-Tg mice but ameliorated in TWEAK-KO mice compared with wild-type mice. Denervated soleus muscle of TWEAK-KO mice showed improved absolute force compared with corresponding wild-type mice. Skeletal muscle of TWEAK-Tg mice also showed increased gene expression of collagen I and collagen III and interstitial fibrosis upon denervation indicating that TWEAK can directly induce fibrosis in skeletal muscle tissues. Similar to cell culture studies, TWEAK stimulated the DNA-binding activity of NF-κB and increased the gene expression of MuRF1 in denervated skeletal muscle (43). Furthermore, starvation-induced loss of skeletal muscle mass was reduced in TWEAK-KO mice compared with wild-type mice (43). Collectively, these studies provide strong evidence that TWEAK/Fn14 system mediates muscle atrophy in catabolic conditions (Figure 2). It is noteworthy that denervation and starvation did not affect the expression TWEAK in skeletal muscle of mice. Similar to studies in rodents, a recent study reported that chronic hemiplegia-induced muscle atrophy was not accompanied with changes in expression of TWEAK in humans (71).

While TWEAK affects the activation of a number of signaling pathways and proteolytic systems in cultured myotubes, only a few of them appear to be regulated by TWEAK in skeletal muscle of mice in atrophic conditions. For example, TWEAK inhibits the activity of PI3K/Akt pathway in cultured myotubes; however, there was no difference in level of phosphorylation of Akt in denervated muscle of wild-type, TWEAK-Tg and TWEAK-KO mice. Furthermore, while denervation increased expression of several autophagy-related genes (such as LC3B, Beclin1, Atg5, Atg12, and Gabarapl1), their expression levels were comparable in denervated skeletal muscle of wild-type, TWEAK-Tg and TWEAK-KO mice (43). These results suggest that denervated skeletal muscle is enriched with certain growth factors, which neutralize some of the actions of TWEAK. However, we cannot rule out that TWEAK/Fn14 signaling also affects the activation of PI3K/Akt and ALS in other conditions of muscle atrophy.

## Regulation of Fn14 Expression in Skeletal Muscle

While, it is increasingly clear that the expression of Fn14 is a rate-limiting step, molecular mechanisms leading to its increased expression in skeletal muscle in atrophic conditions remain poorly understood. Both human and mouse Fn14 promoters lack a typical TATA box, which is generally required for the expression of mammalian genes. However, the promoter region of Fn14 contains consensus sequence for several transcription factors (72). Recent studies in our laboratory have shown that mouse and human Fn14 promoters contain a cytosine–guanine dinucleotide (CpG) island close to transcription start site. Fn14 promoter also contains consensus DNA sequence for AP1 and specificity protein 1 (SP1) transcription factors. Our studies suggest that denervation causes hypomethylation at specific CpG sites in mouse Fn14 promoter in skeletal muscle. Interestingly, while all three DNA methyltransferases (Dnmts) interact with Fn14 promoter in naïve muscle, the levels of DNA methyltransferase 3a (Dnmt3a) are repressed in denervated skeletal muscle of mice. The role of Dnmt3a in Fn14 expression has been supported by our findings that overexpression of Dnmt3a inhibits the expression of Fn14 and attenuates denervation-induced muscle atrophy. We have also found that denervation increases the activation of MAPK, AP1, and SP1 and they are involved in the expression of Fn14 in denervated skeletal muscle.

## TWEAK Promotes Slow-To-Fast Fiber Type Transition in Skeletal Muscle

Rodent skeletal muscle contains four fiber types based on the primary expression of MyHC: I, IIA, IIX, and IIB (73, 74). Type I fibers are slow to twitch by stimuli, display a two to threefold higher mitochondrial content, and rely largely on oxidative metabolism to produce ATP. In contrast, type II fibers are fast in responding to stimuli and rely on glycolytic metabolism as a major energy substrate (75). Type IIB fibers have relatively few mitochondria and store large amounts of glycogen; therefore they are glycolytic in metabolism. Types IIA and IIX fibers have physiologically intermediate properties of slow and fast twitch fibers, which are also rich in mitochondria and possess a relatively high capacity to generate ATP by oxidative metabolism (76, 77). Moreover, it is now increasingly evident that different fiber types display different sensitivity to atrophy stimuli. Oxidative fibers are somewhat resilient to atrophy upon denervation (78) whereas glycolytic fibers undergo more rapid atrophy in response to starvation or sepsis (79, 80).

TWEAK/Fn14 axis appears to be an important regulator of fiber type composition in skeletal muscle. Tg overexpression of TWEAK in mice causes a significant reduction in proportion of type I fibers with a concomitant increase in type II fibers in both soleus and extensor digitorum longus muscle (43). Moreover, hind limb muscle of the founder TWEAK-Tg mice, which could not survive beyond neonatal age, appeared pale compared with wild-type littermates indicating lower myoglobin abundance and fast muscle phenotype in TWEAK overexpressing muscle (47). By contrast, proportion of types I and IIA muscle fibers and skeletal muscle mitochondrial content was found to be increased in skeletal muscle of TWEAK-KO mice (43, 81). These results suggest that TWEAK favors fast-type fiber phenotype even under normal conditions.

**Figure 3:**
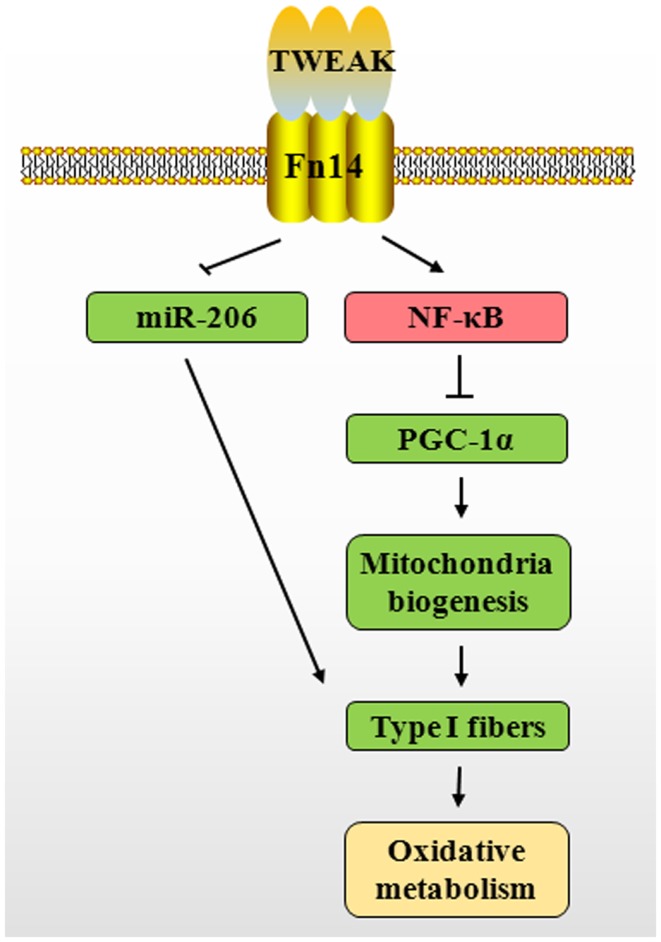
**Putative mechanisms by which TWEAK–Fn14 system inhibits skeletal muscle oxidative phosphorylation capacity**. TWEAK/Fn14 system activates canonical NF-κB signaling pathway, which represses PGC-1α levels in skeletal muscle. This leads to reduced expression of several mitochondrial genes and proportion of type I fibers. TWEAK also decreases the expression of miR-206, a positive regulator of type I fibers. TWEAK, TNF-like weak inducer of apoptosis; Fn14, fibroblast growth factor inducible 14; NF-κB, nuclear factor-kappa B; PGC-1α, peroxisome proliferator-activated receptor-gamma coactivator 1α; miR-206, microRNA-206.

Recent studies have shown that PGC-1α plays an important role in regulating skeletal muscle fiber composition, mitochondrial content, and oxidative metabolism in both physiological and pathophysiological conditions (64, 82, 83). PGC-1α is expressed preferentially in skeletal muscle enriched in type I fibers. Positive role of PGC-1α in inducing type I fibers is supported by the findings that Tg overexpression of PGC-1α in skeletal muscle increases the proportion of types I and IIA fibers (84). Conversely, muscle-specific PGC-1α KO mice exhibited a shift from oxidative types I and IIA toward types IIX and IIB muscle fibers (85) while global PGC-1α KO mice did not show a marked skeletal muscle phenotype (85, 86). PGC-1α drives the transcription of type I muscle fibers through co-activation of myocyte enhancer factor 2 (MEF2) family members such as MEF2c and MEF2d (84). MEF2 and PGC-1α also regulate the expression of PGC-1α in an auto-regulatory loop (87). Interestingly, our recent studies have shown that TWEAK suppresses the expression of MEF2c in C2C12 myotubes (62). Therefore, it is likely that TWEAK increases the number of type II fibers through reducing MEF2/PGC-1α signaling (Figure 3). Supporting this notion, our recent study has demonstrated that transcript levels of PGC-1α and several molecules involved in mitochondrial oxidative metabolism are significantly increased in skeletal muscle of 5-month-old TWEAK-KO mice. Furthermore, treatment of cultured primary myotubes with TWEAK drastically reduced levels of PGC-1α and other mitochondria-related genes (81). Likewise, an inverse relationship between TWEAK and PGC-1α has been observed in denervated skeletal muscle of mice (67). Since type II muscle fibers are more susceptible to atrophy compared to type I muscle fibers in many chronic diseases, it will be interesting to investigate whether slow- to fast-type fiber transition is essential for TWEAK-induced muscle atrophy.

## TWEAK Inhibits Skeletal Muscle Oxidative Metabolism

Skeletal muscle, due to its large mass, is the principal organ for glucose disposal in the body and therefore even a small reduction in skeletal muscle glucose uptake capacity or its ability to metabolize glucose is sufficient to cause metabolic abnormalities and obesity. The decline in skeletal muscle oxidative phosphorylation capacity during aging has been recognized as a major cause for increased fatigability, lowered quality of life, and morbidity (19). The loss of oxidative capacity with elevated levels of proinflammatory cytokines is also implicated in development of metabolic syndrome in many chronic disease states, while the maintenance with exercise has been found to be beneficial to some extent (88, 89).

We have recently studied the role of TWEAK in exercise tolerance and skeletal muscle oxidative phosphorylation capacity. TWEAK-KO mice run longer and with higher speed in a treadmill exercise tolerance test (81). Furthermore, TWEAK-KO mice show augmented levels of subsarcolemmal and intermyofibrillar mitochondria, increased succinate dehydrogenase (SDH)-positive myofibers, and elevated gene expression of metabolic proteins such as PGC-1α, PPARδ, and mCPT-1. Moreover, oxidative phosphorylation is also increased in exercised TWEAK-KO mice compared with wild-type mice. Consistent with studies in TWEAK-KO mice, treatment of cultured myotubes with TWEAK decreased mitochondrial biogenetic capacity and maximal respiratory activity. TWEAK also reduced the expression of PGC-1α and several mitochondrial genes in cultured myotubes (81). Recent studies also suggest that TWEAK represses PGC-1α in cultured cardiomyocytes and myotubes through the activation of canonical NF-κB signaling pathway (67, 90). Collectively, these studies suggest that the repression of PGC-1α is an important mechanism by which TWEAK reduces mitochondrial content and oxidative phosphorylation capacity in skeletal muscle and in other cell types.

There is also a possibility that TWEAK inhibits skeletal muscle oxidative phosphorylation capacity through affecting levels of various miRs. The expression of miRs is sensitive to cytokine levels and alternation of miRs in response to inappropriate cytokine simulation may result in disrupting metabolic homeostasis (91). A subset of muscle-specific miRs has been shown to play an important role in skeletal muscle development and metabolic adaptation (92). Abundance of miR-206 is significantly higher in slow-type skeletal muscle compared with fast-type muscle (93). Levels of miR-206 are diminished during slow-to-fast fiber type transition in response to unloading (94). Furthermore, levels of miR-206 in vastus lateralis have been found to be significantly decreased in patients with type II diabetes compared with healthy individuals (95). Our low-density miRNA array analysis has shown that TWEAK inhibits the expression of miR-206 in C2C12 myotubes (62). These findings suggest that TWEAK may also inhibit skeletal muscle oxidative metabolism through down-regulation of miR-206 levels (Figure 3).

## Concluding Remarks

The studies summarized above indicate that TWEAK/Fn14 system plays an essential role in skeletal muscle remodeling and metabolism. Most of these observations suggest that TWEAK/Fn14 signaling causes loss of skeletal muscle mass and decreases skeletal muscle oxidative metabolism implying that the inhibition of this cytokine-receptor axis can be used as a therapy to maintain skeletal muscle mass and metabolic function. We believe that TWEAK/Fn14 system is among the most attractive drug targets to combat muscle wasting. TWEAK being an extracellular protein *per se*, TWEAK-dependent signaling can be blocked using a TWEAK neutralizing antibody or soluble Fn14-Fc decoy protein, which prevents TWEAK binding to Fn14 cell surface receptors. Indeed, these two reagents have been found to be effective in improving pathologic condition in mouse models of some other diseases where TWEAK/Fn14 signaling is elevated. Alternatively, small-molecule antagonists that prevent Fn14 trimerization or interaction of TWEAK with Fn14 can also be used to block catabolic actions of TWEAK in skeletal muscle.

Whereas the role of TWEAK/Fn14 signaling in skeletal muscle has become increasingly clear, there are still some outstanding questions that need to be addressed. For example, it is important to identify other conditions where TWEAK/Fn14 axis is a mediator of muscle wasting. Furthermore, it is important to investigate the mechanism by which TWEAK causes metabolic abnormalities and whether the inhibition of TWEAK/Fn14 signaling can prevent type II diabetes and obesity in response to high fat diet. It will also be interesting to examine whether TWEAK affects mitochondrial content through regulating mitochondria biogenesis, fusion, fission, or mitophagy. The role of various miRs in regulation of TWEAK-induced muscle atrophy and metabolic dysfunction also needs more investigation. Nevertheless, recent studies have provided strong evidence that TWEAK/Fn14 system is a major regulator of skeletal muscle mass and function.

## Author Contributions

Ashok Kumar conceptualized the study. Shuichi Sato, Yuji Ogura, and Ashok Kumar wrote the manuscript.

## Conflict of Interest Statement

The authors declare that the research was conducted in the absence of any commercial or financial relationships that could be construed as a potential conflict of interest.
